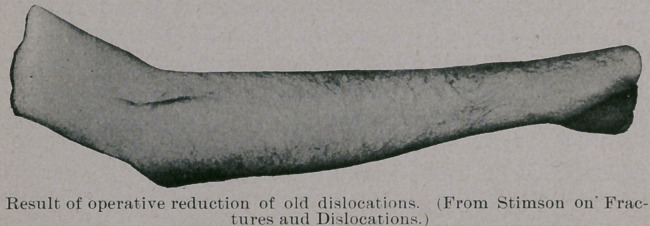# Old Dislocations of the Elbow

**Published:** 1899-03

**Authors:** 


### Old Dislocations of the Elbow.

Dislocations nowhere become inveterate and irreducible sooner than at the elbow. This is especially true in the young, where the developmental osteogenetic power of the periosteum is in full play, and where, consequently, the slightest injury or chronic irritation of the periosteum causes new bone-formation, the presence of which precludes the possibility of the joint surfaces reassuming their old relations. The soft parts, too, in growing individuals are much more easily modified in their development by irritative factors than later in life, so that hindrance to the reduction of a dislocation soon supervenes in the course of a case from faulty evolution of the involved soft tissues. Finally, the ultimate bone relations in joints and the nice correspondence of apposing surfaces are the result of pressure and counterpressure of the parts upon each other during growth, and this being absent, deformity of the bony parts of the joints necessarily follows.

The importance of the movements of the elbow-joint is very great, and, besides, from an æsthetic standpoint, freedom of motion here is very desirable, since limitation of it always causes a striking peculiarity in the holding of the limb and awkwardness in the movement of it that are very noticeable. As stated before, reduction even by force soon becomes impossible. The necessity for early diagnosis and prompt reduction is greatly emphasized. Where inveteracy is once established, if the deformity is considerable, arthrotomy is indicated. The results of operative intervention have frequently in the past, however, been extremely unsatisfactory, and for two reasons: either too little of the abnormal structures that caused persistence of the dislocation were removed,

New formation of bone on an old, unreduced dislocation. (From Stimson on Fractures and Dislocations.)

Result of operative reduction of old dislocations (From Stimson on Fractures and Dislocations.)

Result of operative reduction of old dislocations. (From Stimson on Fractures aud Dislocations.)

in which case inevitably it recurred (often under the operation bandage); or too much of the bony structure was removed, an excision of the elbow being practically done, when a flail joint resulted—an eninently undesirable result.

Professor Stimson, in his new book on "Fractures and Dislocations,"* treats the subject with his well-known practical conservatism. He gives a sketch of new formation of bone on an old, unreduced dislocation of the elbow, as he has seen it in a number of cases. He advises operation for the condition by a long incision on the outer side, exposing the radius and the mass of new bone This should be freely chiselled away and the capitellum exposed by free division of the soft parts, keeping the knife at a little distance from the bone, so as not to damage the periosteum. The sigmoid fossa is then cleared of fibrous tissues. A second incision is now made on the inner side, curving close behind the epitrochlea or its site, the ulnar nerve is drawn forward, and the olecranon freed. If the epitrochlea has been broken off and displaced upward and backward it must be detached from the humerus, preserving its relations with the lateral ligament. The clearing of the sigmoid cavity is then completed. The only obstacle to reduction, then, if there be one, will be the shortening of the flexor muscles of the hand, induced by their action in the abnormal position caused by the dislocation. If necessary they must be partly divided close to the humerus. Professor Stimson gives a picture of one of his results, which we produce. Altogether he has operated upon some ten cases by this method, and the results have all been flexion within a right angle and extension varying from 120 to 170 degrees, with preservation of rotation.

*A Treatise on Fractures and Dislocations, by Lewis A. Stimson, B. A., M. D., Professor of Surgery in Cornell University Medical College, New York. Lea Brothers & Co. Just issued.

## Figures and Tables

**Figure f1:**
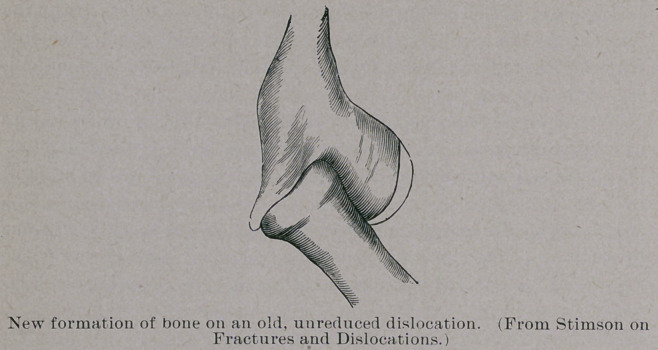


**Figure f2:**
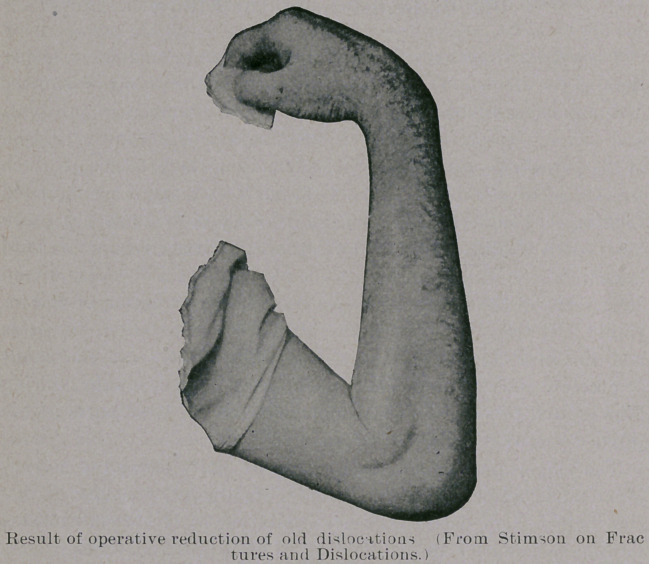


**Figure f3:**